# Supramolecular Recognition of *Escherichia coli* Bacteria by Fluorescent Oligo(Phenyleneethynylene)s with Mannopyranoside Termini Groups

**DOI:** 10.3390/s17051025

**Published:** 2017-05-04

**Authors:** Enrique Arias, Maria Teresa Méndez, Eduardo Arias, Ivana Moggio, Antonio Ledezma, Jorge Romero, Giancarlo Margheri, Emilia Giorgetti

**Affiliations:** 1 Centro de Investigación en Química Aplicada (CIQA), Blvd. Enrique Reyna 140, Saltillo 25294, Mexico; ariasenrike@gmail.com (E.A.); tmendez@uaeh.edu.mx (M.T.M.); antonio.ledezma@ciqa.edu.mx (A.L.); jorge.romero@ciqa.edu.mx (J.R.); 2 Istituto dei Sistemi Complessi Via Madonna del Piano 10, Sesto Fiorentino 50019, Italy; giancarlo.margheri@fi.isc.cnr.it (G.M.); emilia.giorgetti@fi.isc.cnr.it (E.G.)

**Keywords:** phenyleneethynylenes, *Escherichia coli*, SPR

## Abstract

*Escherichia coli* is one the most common bacteria responsible of uropathogenic diseases, which motives the search for rapid and easy methods of detection. By taking advantage of the specific interactions between mannose and type 1 fimbriae, in this work two fluorescent phenyleneethynylene (PE) trimers bearing one or two 4-aminophenyl-α-D-mannopyranoside termini groups were synthesized for the detection of *E. coli.* Three bacterial strains: ORN 178 (fimbriae I expression), ORN 208 (mutant serotype with no fimbriae expression) and one obtained from a local hospital (SS3) were used. Laser Scanning Confocal Microscopy (LSCM) and Surface Plasmon Resonance (SPR) were applied for the interaction studies following two different approaches: (1) mixing the oligomer solutions with the bacterial suspension, which permitted the observation of stained bacteria and by (2) biosensing as thin films, where bacteria adhered on the surface-functionalized substrate. LSCM allows one to easily visualize that two mannose groups are necessary to have a specific interaction with the fimbriae 1. The sensitivity of SPR assays to *E. coli* was 10^4^ colony forming unit (CFU)/mL at 50 µL/min flow rate. The combination of PE units with two mannose groups results in a novel molecule that can be used as a specific fluorescent marker as well as a transducer for the detection of *E. coli*.

## 1. Introduction

*Escherichia coli* is one of the most common bacteria responsible for urinary tract infections (UTIs). Uropathogenic *E. coli* uses type 1 fimbriae for adhering and colonizing the host [1] through specific binding of their top adhesin (FimH) to a high-weight mannose glycoprotein, uroplakin Ia, which is present in the differentiated uroepithelial cells [2,3,4]. Increasing resistance of *Escherichia coli* UTIs has motivated the research of a detection method that could be quicker and easier with respect to classical clinical studies through bacterial growth. Optical biosensors have been proposed using as recognition mechanism this specific interaction and fluorescence or Surface Plasmon Resonance (SPR) spectroscopy as transducing techniques, due to their low limit of detection (LOD).

With respect to fluorescence detection of *Escherichia coli*, conjugated polymers such as poly(phenylene)s [5], poly(fluorene)s [6], poly(phenyleneethynylene)s (PE) [7,8,9,10,11], poly(thiophene)s [12], poly(phenylenevinylidene)s [13] functionalized with mannose or other carbohydrates were reported. All of these works demonstrate the specificity of the molecules to uropathogenic *Escherichia coli* compared to other bacteria or even other *E. coli* strains that do not express type 1 fimbriae. Disney and Bunz [7] also obtained the detection limit of the bacteria by fluorescence microscopy. Despite these excellent results as markers, a real biosensing, i.e., the quantification of a signal towards *E. coli* concentration is not feasible by fluorescence techniques. First, fluorescence intensity depends on the experimental conditions (instrument, excitation wavelength, temperature) and thus a standard and/or a calibration curve should be employed for each specific device. Moreover, the turbidity of a bacteria suspension can affect the determination (*vide infra*). Indeed in the previous works, sensing by fluorescence spectroscopy is limited to a model protein (concanavalin A), or, when *E. coli* bacteria is used, only the quenching of the fluorescence intensity of the conjugated polymer in presence of a certain bacterial number, typically 10^7^–10^8^ CFU/mL equal to O.D. at 600 nm of 0.05–1) is reported.

Surface Plasmon Resonance (SPR) is an alternative technique that is widely used in sensing and biosensing due to the high sensitivity and specificity of detection [14,15]. *Escherichia coli* has been sensed by SPR through different approaches. For example, Waswa et al. [16] modified a SPR gold sensor with biotinylated antibody of *E. coli* achieving a specific detection with a sensitivity of 10^2^–10^3^ CFU/mL for *E. coli* O157:H7 that is an important food contaminant enteric pathogen. Other authors used carbohydrate-modified SPR chips; for instance Finne et al. detected P-fimbriated uropathogenic *E. coli* by SPR modifying a sensor chip with galabiose-BSA conjugate and investigated the inhibition of this adhesion with multivalent galabiose derivatives [17]. J. Bouckaert reported the functionalization of gold sensors with aminoheptyl α-D-mannopyranoside for uropathogenic *E. coli* detection by SPR [18]. Aromatic α-D-mannosides have been demonstrated to present improved binding properties with respect to aliphatic ones because the aromatic group interacts with the hydrophobic binding region that is located at FimH binding domain [19]. Following this strategy, fullerene adducts bearing 12 peripheral mannose groups were also studied by SPR (and other techniques) as inhibitors of FimH [20].

Based on this background, we report in this paper the synthesis of two phenyleneethynylene oligomers bearing one or two mannose termini groups that could allow the specific detection of uropathogenic *Escherichia coli* by fluorescence microscopy and its biosensing by Surface Plasmon Resonance showing a LOD of 10^4^ CFU/mL of *E. coli* at a flow rate of 50 µL/min. The results herein reported reveal that the combination of the PE unit with mannose groups affords fluorescent molecules that can be used as both specific markers and transducers for biosensing of type 1 fimbriated *E. coli* by supramolecular interaction, and can be fruitfully exploited to engineer an optimized biosensing chip for its specific recognition.

## 2. Experimental Details

### 2.1. Materials and Methods

All the reagents were purchased from Sigma-Aldrich (Sigma-Aldrich Química, Toluca, Mexico). UV-Vis and fluorescence spectroscopy were carried out on a 2401 PC (Shimadzu Scientific Instruments Inc, Columbia, MD, USA) or a LS50B (Perkin Elmer, Mexico City, Mexico) instrument, respectively. For fluorescence spectra, excitation wavelength was chosen at 10 nm under the absorption lower energy peak and the experiments were carried out at 25.0 ± 0.5 °C with a water circulating bath. The quantum yield was obtained according to [21]. Quinine sulfate in 0.1 M H_2_SO_4_ was used as standard. Five repetitions were realized and the average value is reported in the corresponding table.

### 2.2. Synthesis

Experimental procedures, chemical and physicochemical characterization of each compound are given in the Electronic Supplementary Information (ESI) section.

### 2.3. Bacteria Microscopic Characterization and Agglutination Assays

Experimental details on the bacteria growth, microscopic characterization and agglutination assays are reported in the ESI.

### 2.4. Recognition Assays By LSCM

Laser Scanning Confocal Microscopy was carried out with a Pascal 5 instrument from Carl Zeiss (Mexico City, Mexico), in a two channel (fluorescence and reflection) mode. The excitation was obtained with an Ar laser (480 nm, 200 mW). 3 mL of distilled water were added to 100 μL of the *E. coli* suspension prepared as mentioned and the bacterial suspension was verified under an optical microscope. Then, 100 μL of 1 g/L NMP solution of the PE oligomers were added and the mixture was shaken and then incubated for 40 min at 37 °C and 120 rpm. After that, two centrifugation cycles with water and then with NMP were applied in order to eliminate any residual oligomer that did not interact. Finally 10 μL of the suspension were placed on a microscope slide with a platinum handle and left to dry at room temperature in a sterile laminar flow hood.

### 2.5. SPR Tests

The sensing platforms were SF4 glass slides, with laser-grade polished surfaces, covered with an aluminum layer that was deposited with a high vacuum, electron-gun assisted facility (evaporation rate: 0.1 nm/s), with no pretreatment of the glass surfaces.

The sensor chip was characterized with a previously described homemade SPR spectrometer [22], working in the classical Kretschmann configuration, operating at the wavelength of 633 nm. Briefly, the instrument can measure the reflectivity of the chip vs the rotation angle, obtaining the so called SPR angular spectrum or, alternatively, measure the reflectivity at a fixed incidence angle, to obtain a time varying signal that resembles the so called sensorgram of the binding process.

The functionalization of the sensing surface was carried out by drop casting the oligomer on the metallized glass from a 1 g/L solution in DMF: THF (95:5 *v*/*v*) and then left in a freeze dryer system (Labconco Corporation, Kansas City, MI, USA). The functionalization was verified by LSCM and by measuring the SPR spectra of the covered sample. The experimental SPR spectra of the bare and functionalized chips have been fitted with Winspall 2.0 software (Res-tech, Framersheim, Germany) in order to get the thickness and refractive index of the layer (s).

The sensing performances were tested in two steps, first by monitoring the ability to bind the type 1 fimbriae, a mandatory prerequisite for the bacteria recognition, and, subsequently, by checking the capability to recognize the ORN 178 *E. coli* strain. A negative control test was finally performed using ORN 208 non fimbriated bacteria. The biological samples were prepared as mentioned in the ESI.

The first step, namely the capture of fimbriae, was carried out with the homemade instrument, while the tests on bacteria were performed by recording sensorgrams with a commercial SPR instrument (Reichert 7000, Depew, NY, USA) that utilizes 633 nm wavelength laser light as excitation source, in dynamic conditions under a constant flow of 50 μL/min. 250 μL of the ORN 178 *E. coli* suspension prepared as previously described were injected at three cells concentrations (10^4^, 10^5^, 10^6^ CFU/mL), with water rinsing in between each injection. The control test was performed using a dispersion of ORN 208 with concentration of 10^5^ CFU/mL.

## 3. Results and Discussion

### 3.1. Synthesis and Characterization of the α-D-Mannosides Substituted Phenyleneethynylenes

**Scheme 1:**
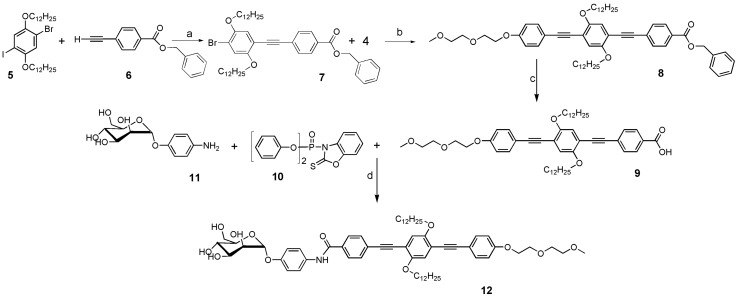
Reagents and conditions: (a) [(C_6_H_5_)_3_P]_2_PdCl_2_ (2.5 % mol), CuI (1.5 % mol), Et_3_N, 0 °C -to-rt, 18 h, (65%); (b) [(C_6_H_5_)_3_P]_2_PdCl_2_ (2.5 % mol), CuI (1.5 % mol), Et_3_N, 60 °C, 1 h, (70%); (c) KOH, toluene, N_2_, 110 °C, 2 h (87%); (d) 1-methyl-2-pyrrolidone , Et_3_N, rt, 96 h, (75%).

**Scheme 2:**
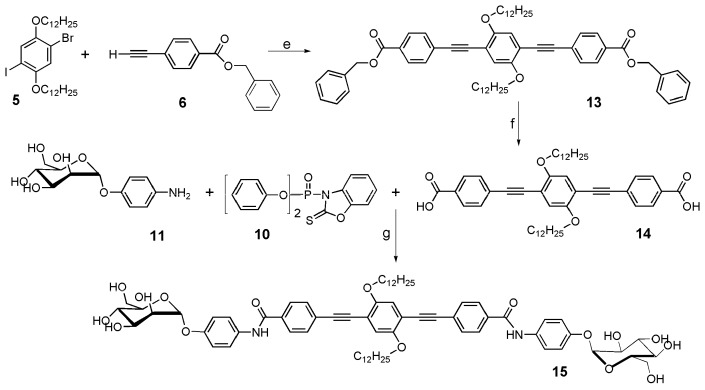
Reagents and conditions: (e) [(C_6_H_5_)_3_P]_2_PdCl_2_ (2.5 % mol), CuI (1.5 % mol), Et_3_N, 60 °C, 18 h, (87%); (f) KOH, toluene, N_2_, 110 °C, 2 h (90%); (g) 1-methyl-2-pyrrolidone , Et_3_N, rt, 96 h, (77%).

Two α-D-mannoside-substituted phenyleneethynylene trimers were synthesized: one bearing a glycol and a mannopyranoside group at each extremity, and the other one with two mannopyranoside groups at both extremities. In both oligomers, two dodecanoxy chains were introduced in the central phenyl ring to promote solubility. The pathway for their synthesis is depicted in Scheme 1 and Scheme 2.

In most of the works related with oligomers or polymers substituted with mannose [23,24,25], the OH groups of the carbohydrate must be previously protected by acetylation, and subsequently subjected to deacetylation with the tedious work up to isolate the different percentage of deacetylated products. Furthermore, the presence of other functional groups in the molecule susceptible to the hydrolysis conditions must be taken into account. Here, we first prepared step-by-step oligomers having three phenyleneethynylene units and terminated with one (**9**, Scheme 1) or two (**14**, Scheme 2) benzyl-protected carboxylic acids by Pd/Cu cross-coupling [26] of monomer **5** [27] with one or two equivalents of **6** to give the bromo-terminated dimer **7** [28] that was then Pd/Cu cross-coupled with **4** (Scheme S1) to give the carboxylic acid benzyl-protected trimer **8** or the symmetric benzyl-protected terminated oligomer **13** (Scheme 2). After evaluating different methods and conditions to cleave the benzyl group and to carry out the amidation reactions (ESI), the acid groups were deprotected by using KOH and toluene under nitrogen atmosphere in yields higher than 85% and without the detection of any traces of oxidized ethynylene groups. The carboxylic acids were then activated for the amidation reaction with (2,3-dihydro-2-thioxo-3-benzoxazolyl)-phosphonate (DTBP, **10**), which is a good leaving group after the nucleophilic attack of the amine of the 4-aminophenyl-α-D-mannopyranoside. By this amidation method, neither the primary nor the secondary alcohols have to be protected and remain unperturbed.

The more characteristic signals that confirm the amidation in both compounds are: (i) the resonance peaks of the -NH- group that appear at 10.27 ppm, (ii) those belonging to the anomeric proton at 5.33 ppm and (iii) those of the neighbors to the anomeric at 3.82 ppm. The oligomers are soluble only in very polar solvents such as DMSO, NMP and DMF, solvents that are miscible with water where staining assay can be performed.

**Figure 1:**
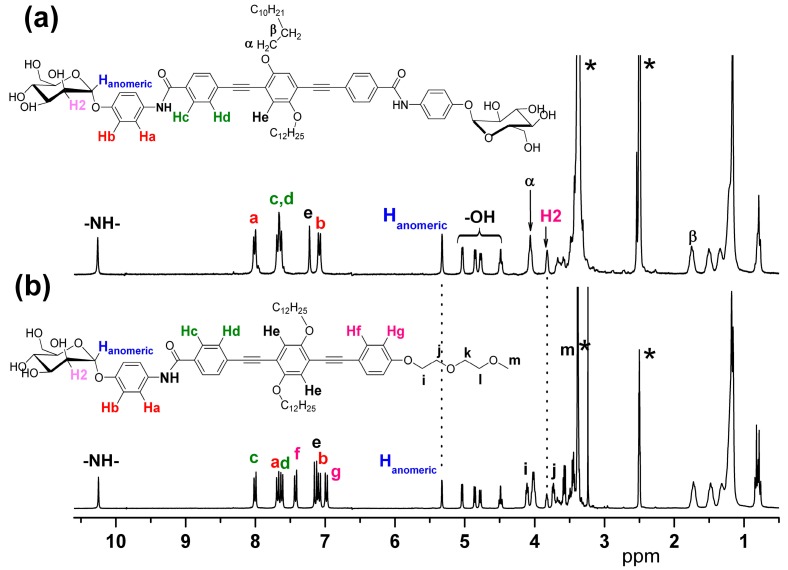
^1^H-NMR spectra in d_6_-DMSO of α-D-mannopyranoside terminated trimers: (**a**) **15** and (**b**) **12**. The multiplicity assignation is addressed to the more representative resonant peaks (asterisks denote d5- DMSO and H_2_O in the samples).

Figure 1 shows the ^1^H-NMR spectra of **12** and **15** in d_6_-DMSO.

### 3.2. Optical Properties in DMF

**Figure 2:**
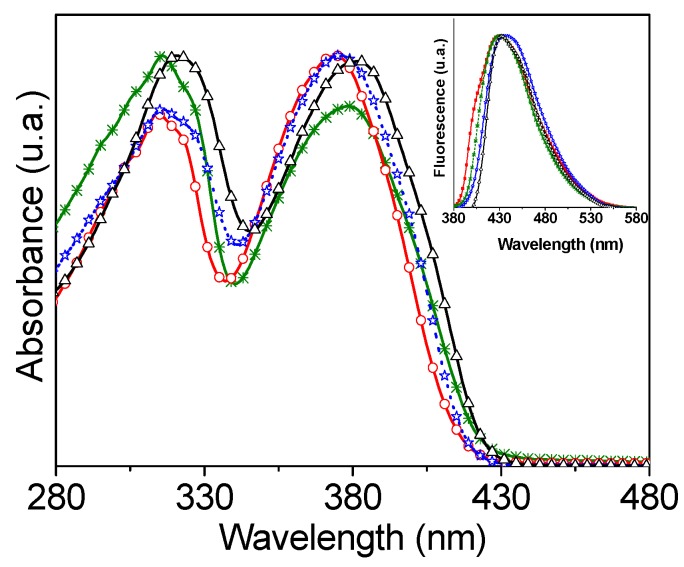
UV-Vis and (inserted) fluorescence spectra of **9** (**triangles**), **12** (stars), **14** (asterisks), and **15** (circles) in DMF.

In Figure 2, the UV-Vis and fluorescence spectra in DMF of the mannose substituted trimers **12** and **15** as well as their carboxylic acid (**9** and **14**) precursors are reported. All of the optical data of these oligomers are collected in Table 1, together with those of the benzyl-protected oligomers **8** and **13** for sake of comparison.

The electronic absorption spectra are characterized by two peaks at around 320 and 380 nm. According to previous studies on hexyloxy-substituted phenyleneethynylenes [29], the peak at 320 nm can be associated with the HOMO-1→LUMO electronic transition, while the lower energy peak is associated with the HOMO→LUMO electronic transition. The maxima of these peaks do not change significantly from one trimer to the other as the differences in the chemical structures of the four are just the termini groups and not the main conjugated backbone. Accordingly, the emission maximum is almost at the same wavelength, around 430 nm. The fluorescence quantum yield (φ) is, however, markedly affected by the termini groups. The acid oligomers present higher φ than the mannose substituted ones, which can be ascribed to the presence of quenching groups (OH and NH) of the aminophenylmannopyranoside.

### 3.3. E. coli Propagation and Fimbriae I Characterization

**Figure 3:**
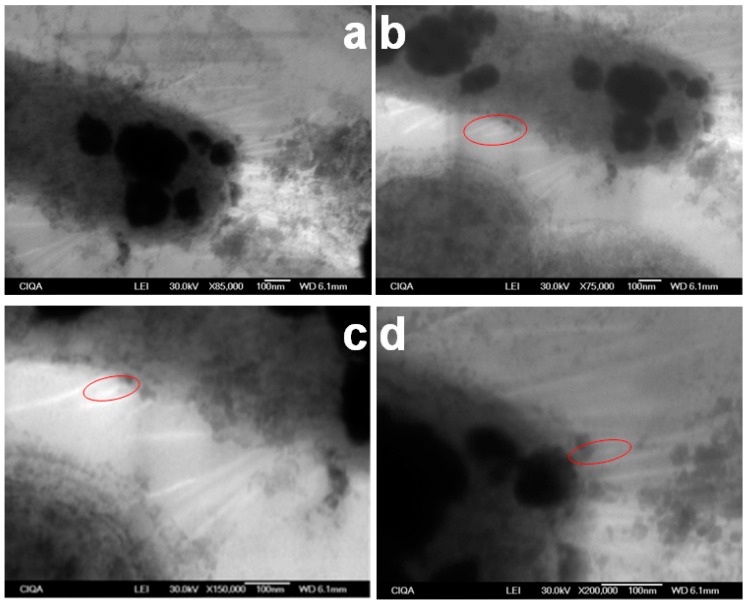
TEM images of the SS3 *E. coli* from a local hospital. Image (**a**) shows the appendages of the bacteria, while images (**b**–**d**) show the fimbriae 1 (red circles).

In this work we obtained an uropathogenic *E. coli* serotype from a local hospital, which we named SS3. As controls, we worked with two bacterial strains [30] kindly donated by Prof. Orndorff, (College of Veterinary Medicine at North Carolina State University); the ORN178 is a positive phenotype for the type 1 fimbriae expression, and the ORN208 that does not express fimbriae 1 as negative control. After following the propagation protocol, and thanks to the fact that fimbriae 1 of *E. coli* bacteria have been largely characterized by AFM and TEM microscopy, it was thus easy to identify them on SS3 by TEM microscopy, Figure 3, while all of the appendages could be clearly seen by SEM microscopy (Figure S1). The fimbriae 1 in SS3, Figure 3b–d, are in the range of 7.0 nm diameter as stated in other works [31,32,33].

The presence of fimbriae 1 on the *E. coli* stains was also corroborated by agglutination tests with *S. cerevisiae*. It was found that both the SS3 and the ORN178 agglutinated the yeast after grown under all conditions (Figure S2) and the agglutinations could be inhibited with 4-aminophenyl-α-D-mannopyranoside **11**, Figure S3. In contrast, ORN 208 did not agglutinate, as expected (Figure S3).

### 3.4. Recognition Assays by LSCM

The fluorescence quantum yield φ is too low to permit *E. coli* biosensing by fluorescence, however, it is sufficient to use the two mannose-functionalized oligomers as markers to recognize the *E. coli* Fim H by LSCM.

**Figure 4:**
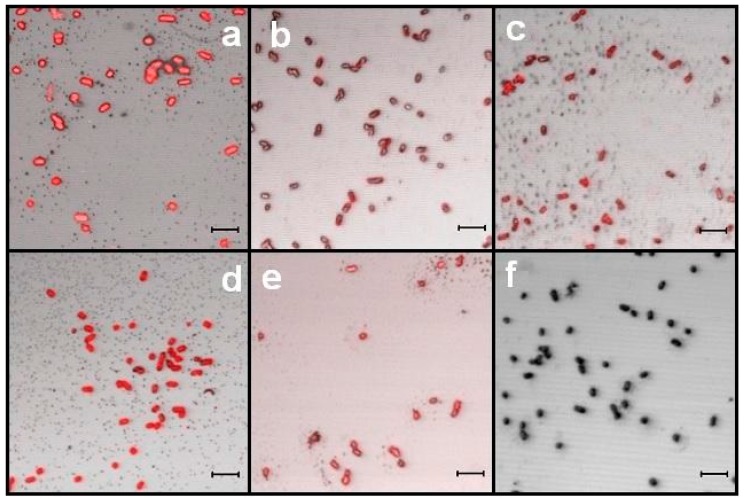
LSCM images of mannose-functionalized oligomers with *E. coli* cells of different serotypes: (**a**) **12** + SS3; (**b**) **12** + ORN178; (**c**) **12** + ORN208; (**d**) **15** + SS3; (**e**) **15** + ORN178; (**f**) **15** + ORN208. Scale bar corresponds to 5 µm.

Figure 4 shows the LSCM images of *E. coli* cells of different serotypes (SS3, ORN178 and ORN208) after adding oligomers **12** and **15**. Each image is the combination obtained directly from the instrument software between the fluorescence (colored image) and the reflection (gray image) detection. Fluorescence comes from the oligomer so that the colored part of the image corresponds to the cells that have been stained. The fluorescence color was selected as red in the palette available of the software, just for enhancing the contrast between the stained and not stained sample even if the emission color of the PEs is blue.

**Figure 5:**
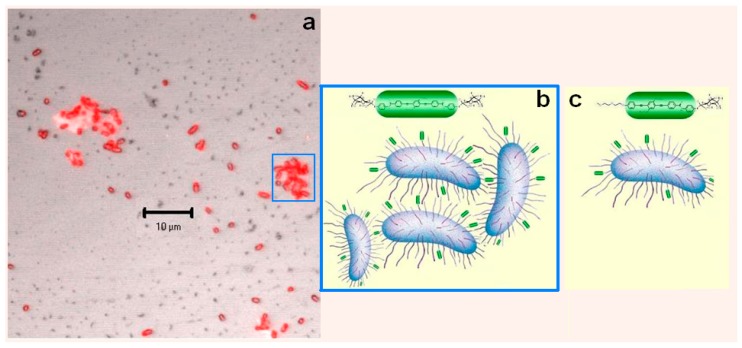
(**a**) LSCM image of a positive agglutination test of *E. coli* SS3 with the oligomer bearing two mannoses **15**; (**b**) sketch of the possible supramolecular recognition: *E. coli*-oligomer **15**; and (**c**) sketch of the *E. coli* bacteria staining with oligomer **12**.

The presence of the *E. coli* typical cells can be observed in all of the figures without any apparent cell damage as a consequence of the preparation. The two oligomers efficiently stain both SS3 and ORN178 *E. coli* bacteria, which likely indicates that the mannose is specific for the fimbriae 1 of these strains. However, **12** also stains the mutant ORN208, which does not express these fimbriae. As a further control, a PE trimer bearing glycols as lateral substituents of the phenyls (3BEgly-H, [34]) was also investigated giving the same results: the three strains were stained (ESI). As all the samples were washed several times in order to rinse any residual oligomer that could just be physically absorbed on the bacteria, this result means that even if the mannose group of **12** could interact with fimbriae 1, the glycol moiety likely promotes the interaction with other flagella or to the bacteria membrane, so that the recognition of this trimer is not specific for uropathogenic *E. coli*. On the contrary, oligomer **15** that has the two mannose groups, does not stain the mutant ORN208 as is evident from Figure 4f where bacteria are not fluorescent. As a further confirmation, it is important to notice that at lower magnification, together with the isolated bacterial cells, we found that both SS3 and ORN178 *E. coli* agglutinate in numerous small packages of cells only with the two mannoses terminated oligomer **15**, Figure 5a. From all these results, we can conclude that the oligomer **15** is specific for fimbriated 1 *E. coli* thanks to the supramolecular recognition between the two mannose groups and the FimH, while oligomer **12** just stains the cells as sketched in Figure 5b,c, respectively. Oligomer **15** was thus selected for uropathogenic *E. coli* biosensing.

### 3.5. SPR Assays

Although we obtained visual evidence of the oligomer **15**-*E. coli* selective interaction by LSCM, a quantitative evaluation using the oligomer’s fluorescence was hampered by two main bottlenecks: (i) the quantum yield of **15** is very low, as previously discussed and (ii) the suspension of *E. coli* is turbid and acts as an optical barrier for the excitation light and fluorescence output as well. Surface Plasmon Resonance [35,36,37] was thus advanced as an alternative spectroscopic technique to develop an optical biosensor aimed at getting quantitative data about the bacteria recognition, primarily the limit of detection (LOD) of their concentration. As planned, our sensing chips have been fabricated by using aluminum as plasmon-supporting metal instead of the most commonly used gold [38,39] because of the higher affinity of **15** to this substrate. Considerations about the chip preparation conditions are reported in ESI.

**Figure 6:**
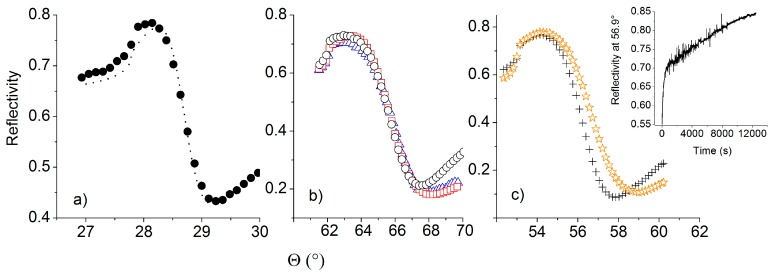
Evolution of SPR curves during the *E. coli* assay. (**a**) Al deposition: experimental values (full circles) and fitted curve (dotted line); (**b**) Oligomer deposition and stability: Al in DMF (open circles), oligomer deposition as prepared (squares) and after 2 washings with DMF (triangles); (**c**) Fimbriae I detection: Al/15 film before (crosses) and after (stars) 15 h of contact with fimbriae I of *E. coli*. Inserted figure: kinetic curve of the interaction between fimbriae 1 of *E. coli* and 15 coated Al sensor.

In the following steps, we characterized via SPR a typical metallized sample using air as substrate (Figure 6a), its functionalization with oligomer **15** (Figure 6b) and, finally, the binding process of type 1 fimbriae on its surface (Figure 6c).

The experimental plot of the reflectivity of Figure 6a (full circles) has a minimum R_min_ = 0.43 at an angular position Ɵ_P,air_ = 29.23°, which fits well only by modeling the experimental points distribution with the reflectivity of a bilayer composed by aluminum (25 nm, refractive index of 1.37 [40]) and alumina (4.5 nm, refractive index of 1.77 [41]). Although the typical value of the alumina layer after an air exposure of 30 min is around 3 nm, nevertheless values up to 4 nm (close to our value) have been reported by Cairns et al. [42] after prolonged exposures. It is worth to notice that the minimum reflectivity, recorded at Ɵ_P,air_, 0.43, indicates a non-optimal thickness of the metal. Indeed, our simulations show that a smaller thickness of 16 nm would be necessary to optimize the coupling efficiency and the figure of merit (FOM, for its definition see for instance [43]) of the sensor. This thickness reduction would allow a 2-fold enhancement of the sensing resolution.

After functionalization of the sensor surface with oligomer **15**, the SPR curve (Figure 6b) in DMF (open circles), shows a resonance angle Ɵ_P,DMF_ of 67.55°, that shifts to 67.96° after the oligomer deposition and rinsing (squares). The increase of 0.41°corresponds to the formation of an oligomer layer whose average thickness is 2.8 nm. It is worth to notice that, in particular in the lower part of the traces, the spectrum of the coated Al/alumina bilayer is wider than that one before functionalization, an effect attributable to the non-uniform surface coverage [44] as observed via LSCM (Figure S6). No significant changes are produced in the spectrum by the second washing (triangles), revealing a good stability of the receptors layer.

**Figure 7:**
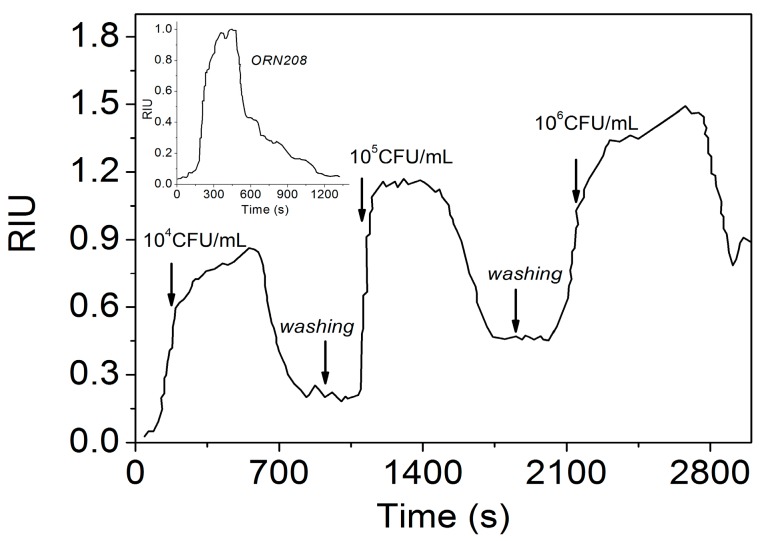
SPR response of **15** coated Al sensor to *E. coli* ORN 178 with different CFU/mL concentrations. Inserted figure: control experiment: SPR response to *E. coli* ORN 208 (10^5^ CFU/mL).

Subsequently, the SPR monitoring of type 1 fimbriae adsorption was carried out, with the measurement sequences reported in Figure 6c. As a first step, we put in contact the sensor surface with the sole liquid environment in which fimbriae 1 are suspended, namely pure water/Ringer liquid mixture (crosses). We observed a resonance angle of 57.70°, lower than that recorded with DMF because of the decrease in the refractive index of the medium. Then, the sensor was exposed to a suspension of fimbriae 1 in water/Ringer solution. After 15 h incubation, the resonance angle shifts to 58.90° (stars) and remains stable even after six washing cycles, indicating a true and effective binding of fimbriae to oligomer **15**, a necessary prerequisite for a successful recognition of the bacterium. The inset of the same panel shows the increase of the reflected signal recorded at an incidence angle corresponding to the flex point of the decreasing flank of the spectrum (56.90°), as a consequence of its right shift. SPR biosensing was then performed with ORN 178 *E. coli* with CFU/mL between 10^4^ and 10^6^ (Figure 7).

In the three kinetic curves of Figure 7, the presence of steady signals obtained after the rinsing cycles indicates effective mass increases atop the sensor, as result of a stable binding of the bacteria onto the surface. Experiments performed with bacteria concentrations lower than 10^4^ CFU/mL did not give appreciable response. This latter concentration was thus established as the LOD of our sensing equipment. This value is satisfactorily comparable to those reported by other authors [45,46]. For instance, in [45] a value of 10^4^ CFU/mL is reported for lysed samples, while this value increases to 10^5^ CFU/mL for heat killed samples and even to 10^6^ for untreated samples. In [46], a detection limit for *E. coli* of 1.2 × 10^4^ CFU/mL was found, while in [47], a LOD of 10^3^ CFU/mL is reported using a detection area functionalized with T4 bacteriophages. In this latter case, however, the better LOD was obtained at expenses of a complex amplification stage of the T4 phages. Even if the LOD of our sensor is comparable with the best results reported, there is still room to manoeuver for its enhancement. For instance, the aforementioned adjustment of the metal thickness can determine a 2-fold increase of the sensitivity, while a proper choice of the radiation wavelength in the blue-UV region can allow the exploitation of more appropriate refractive indices of aluminum. Moreover, the fluorescent emission of the new oligomers could also be fruitfully used in synergy with the enhanced plasmonic field in a surface plasmon enhanced fluorescence configuration, with considerable rise in the sensitivity [48]). The sensor response can be further increased by exploiting more favorable working conditions, as reported in the case of injection flow rate [19] that can significantly influence the binding of *E. coli* to SPR sensors. It is worth mentioning that the SPR assay with the wide strain SS3 (Figure S7) presents a LOD of 10^6^ CFU/mL. This discrepancy can be explained considering that: (a) as SS3 is a wild type strain, the amount of fimbriae structures per bacterium are in a minor proportion; (b) the affinity of Fim H to the α-mannopyranoside oligomer in ORN 178 strain could be much higher than that of the SS3 strain.

As far as selectivity is concerned, a control test has been performed by incubating non-fimbriated bacteria ORN 208 for 400 s with a functionalized sensing chip and recording the kinetics during the incubation and after rinsing. After rinsing, no significant response was obtained, as shown by the inset kinetic curve of Figure 7. In comparison, the incubation of ORN178 bacteria at the same concentration produced a signal that, after rinsing, settled down to ~0.4 RIU, suggesting a good selectivity of the sensor for fimbriated bacteria.

## 4. Conclusions

In this work we reported on the synthesis of two novel phenylenethynylene trimers bearing one or two 4-aminophenyl-α-D-mannopyranoside termini groups for the detection of *E. coli* bacteria*.* Despite the low fluorescence quantum yield, Laser Scanning Confocal Microscopy allowed to demonstrate that both trimers stain *E. coli*, but only the oligomer that bears two mannose groups can discriminate between uropathogenic (type 1 fimbriae generating strain) and the non-uropathogenic *E. coli* mutant strain. Thanks to the good affinity of this trimer to bind stably to the external surface of oxidized aluminum, SPR biosensing of uropathogenic *E. coli* was then performed by fabricating aluminum-based biochips coated with this trimer achieving a LOD of 10^4^ CFU/mL. This study demonstrated a novel SPR *E. coli* bacteria sensing tool that exhibits good performances in LOD and selectivity, together with a noticeable robustness. Considering the number of ways still exploitable to enhance its performance, we think that our findings can pave the way to the realization of a new family of sensitive and selective biochips to detect *E. coli*, whose optimization will be the object of dedicated future works.

## Figures and Tables

**Table 1:** Optical data of the phenyleneethynylenes (PEs) studied in this work in DMF.

PEs	λabs (nm)	ε 10^4^ (M^−1^ cm^—1^)	λemiss (nm)	φ
8	380	7.92	457	0.89
9	314,374	2.65	430	0.94
12	316,376	2.14	438	0.05
13	385	4.98	450	1.00
14	316,378	1.84	430	0.67
15	321,381	9.12	432	0.02

## Supplementary Materials

The following are available online at http://www.mdpi.com/1424-8220/17/5/1025/s1, Experimental synthetic procedures, *E. coli* microscopic characterization, agglutination tests and additional LSCM images. SPR chip preparation and additional SPR curves.
