# Supplementary material for: Supramolecular Recognition of Escherichia coli Bacteria by Fluorescent Oligo(Phenyleneethynylene)s with Mannopyranoside Termini Groups
Source: Sensors (Basel). 2017 May 4;17(5):1025. doi: 10.3390/s17051025 (PMC5469630; doi:10.3390/s17051025)
Supplement: Supplementary file 1 [file sensors-17-01025-s001.pdf]

Supplementary Materials: Supramolecular Recognition of *Escherichia coli* Bacteria by Fluorescent oligo(phenyleneethynylene)s with Mannopyranoside Termini Groups

Enrique Arias, Maria Teresa Méndez, Eduardo Arias, Ivana Moggio, Antonio Ledezma, Jorge Romero, Giancarlo Margheri and Emilia Giorgetti

I. Experimental Synthetic Procedures

Most of the chemical products were obtained from Aldrich, Sigma or Fluka and used without further purification, e.g., 4-aminophenyl α-D-mannopyranoside, copper (I) iodide, dichlorobis(triphenylphosphine) palladium (II), 1-bromo-2-(2-methoxyethoxy) ethane, 4-iodophenol, dicyclohexylcarbodiimide (DCC), dimethylaminopyridine (DMAP), trimethylsilylacetylene (TMSA) and tetrabutylammonium fluoride ($F^-Bu_4N^+$). All of the solvents were purchased from Aldrich, Fluka and Baker. THF and triethylamine (Et_3N) were distilled from KOH and sodium/benzophenone complex, respectively.

The monomer for oligomerization was synthesized in 3 steps (Scheme S1). The 4-iodophenol **1** was alkylated with 1-bromo-2-(2-methoxyethoxy) ethane under the Williamson reaction conditions to obtain **2**. Then, **2** was Pd/Cu cross-coupled with TMSA in triethylamine to obtain **3** in 75% yields, which later undergoes desilylation with $F^-Bu_4N^+$ to give **4** in 91% yields.

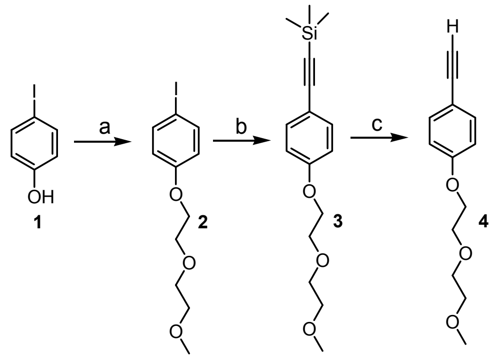

Scheme S1. Reagents and conditions: (**a**) CH_3-(O-CH_2-CH_2)$_2$-Br, NaOH, DMF, 120 °C, 16h, (66%); (**b**) $[(C_6H_5)_3P]_2PdCl_2$ (2.5 % mol), CuI (1.5 % mol), TMSA, Et_3N, 60 °C, 18h, (75%); (**c**) $F^-Bu_4N^+$, THF, rt, 5 min, (91%).

Monomers **1** and **2** were synthetized as reported in the literature [1,2]

General procedure for the Sonogashira cross-coupling of an acetylene with a bromo- or iodoaryl. A two neck round bottomed flask containing previously degassed Et_3N (and ~5% of THF in the Godt's procedure for iodoaryls) is charged with $[(C_6H_5)_3P]_2PdCl_2$, CuI and the bromoaryl (or iodoaryl) under nitrogen. The mixture is heated at 50 °C for 15 min (by the Godt's method for iodoaryls after heating, it is then cooled at ~0 °C). Then, the acetylene monomer is added under nitrogen atmosphere and stirred vigorously overnight at 70–80 °C (by the Godt´s method after adding the acetylene, the reaction is left to reach room temperature and stirred for 48 h). The mixture is filtered off to eliminate the ammonium salt,

washed with THF and later the solvent is evaporated. The crude product is ready for the next purification step.

General procedure for the desilylation. $F^-Bu_4N^+$ (0.2 equivalents per silyl group, 1 M solution in THF) is added into a round bottomed flask charged with the (trimethylsilyl)ethynyl compound, THF and two drops of water. The mixture is stirred at room temperature for 30 min and then the reaction is stopped by pouring it into a dry plug of silica gel. After eluting with THF, the solvent is evaporated and the product is dried in vacuum for two hours and then used without further purification.

1-iodo-4-(2-(2-methoxy ethoxy)ethoxy)benzene (**2**). In a 250 mL bottomed flask, equipped with magnetic stirrer, and condenser were added 2 g (9.09 mmol) of 4-iodophenol **1**, 0.545 g (14 mmol) of NaOH and 1.66 g (1.36 mL, 9.09 mmol) of 1-bromo-2-(2-metoxi-etoxi)-ethane in 15 mL of DMF. The reaction mixture was stirred and heated at 120–130 °C for 16 h. After cooling, 30 mL of cold water were added and the organic phase extracted with 50 mL of CH_2Cl_2, dried with $MgSO_4$, filtered and the solvent evaporated. The crude product was purified by silica gel column chromatography ($CHCl_3$/acetone, 95/5 v/v, rf = 0.6) to obtain a pale pink liquid in 66 % yield (1.93 g). ^{1}H NMR (300 MHz, $CDCl_3$) δ(ppm) 7.26 (d, 2H, H2, H6), 6.42 (d, 2H, H3, H5), 3.76 (t, 2H, -CH_2-α-O-Ar), 3.53 (t, 2H, -CH_2-β-O-Ar), 3.40 (t, 2H, -CH_2-β-O-CH_3), 3.27 (t, 2H, -CH_2-α-O-CH_3), 3.09 (s, 3H, CH_3-O-). ^{13}C NMR (300 MHz, $CDCl_3$) δ(ppm) 83.02 (C1), 138.09 (C2, C6), 117.08 (C3, C5), 158.65 (C4), 71.88 (C-α-O-CH_3), 70.62 (C-α-O-Ar), 69.50 (C-β-O-CH_3), 67.46 (C-β–O-Ar), 58.95 (-CH_3).

4-(2-(2-methoxy ethoxy)ethoxy)-trimethylsylilethynylenbenzene (3). Applying the procedure for the Sonogashira cross-coupling: in a two neck flask containing distilled and degassed Et_3N/THF (15 mL/5 mL) were added under nitrogen 131 mg (0.186 mmol) of diclorobis(triphenyl) phosphine palladium II, 17 mg (0.093 mmol) of CuI and 2000 mg (6.21 mmol) of **10**. The mixture was heated at 50 °C for 10 min and then cooled at 0 °C. Later 609 mg (0.88 mL, 6.21 mmol) of TMSA was added by syringe. The mixture was left to reach room temperature and to react for 48 h. After filtering the ammonium salt, the solvent was evaporated and the crude product was purified by column chromatography (SiO_2, $CHCl_3$, rf = 0.6) to obtain a brown viscous liquid, 62% yield (1.13 g). ^{1}H NMR (300 MHz, $CDCl_3$) δ (ppm) 7.35 (d, 2H, H2, H6), 6.78 (d, 2H, H3, H5), 4.07 (t, 2H, -CH_2-α-O-Ar), 3.81 (t, 2H, -CH_2-β-O-Ar), 3.66 (t, 2H, -CH_2-β-O-CH_3), 3.52 (t, 2H, -CH_2-α-O-CH_3), 3.34 (s, 3H, CH_3-O-), 0.2 (s, 9H, $(CH_3)_3$-Si). ^{13}C NMR (300 MHz, $CDCl_3$) δ (ppm) 115.38 (C1), 133.38 (C2, C6), 114.46 (C3, C5), 158.97 (C4), 105.32 y 92.32 (C≡C),71.88 (C-α-O-CH_3), 70.63 (C-α-O-Ar), 69.56 (C-β-O-CH_3), 67.35 (C-β-O-Ar), 58.91 (-CH_3), -0.12 (($CH_3)_3$-Si).

1-ethynyl-4-(2-(2-methoxy ethoxy)ethoxy)benzene (4). Applying the procedure for the desilylation: a dark brown liquid in 91% yield (670 mg) is obtained. ^{1}H NMR (300 MHz, $CDCl_3$) δ (ppm) 7.33 (d, 2H, H2, H6), 6.77 (d, 2H, H3, H5), 4.03 (t, 2H, -CH_2-α–O-Ar), 3.75 (t, 2H, -CH_2-β-O-Ar), 3.62 (t, 2H, -CH_2-β–O-CH_3), 3.48 (t, 2H, -CH_2-α-O-CH_3), 3.30 (s, 3H, CH_3-O-), 2.97 (s, 1H, H-≡). ^{13}C NMR (300 MHz, $CDCl_3$) δ (ppm) 114.32 (C1), 133.49 (C2, C6), 114.59 (C3, C5), 159.15 (C4), 83.69 y 76.39 (C≡C), 71.86 (C-α-O-CH_3), 70.65 (C-α-O-Ar), 69.52 (C-β-O-CH_3), 67.40 (C-β-O-Ar), 58.90 (-CH_3).

Benzyl 4-((4-bromo-2,5-bis(dodecanoxy)phenyl)ethynyl)benzoate (7). Applying the procedure for the Sonogashira cross-coupling: in a two neck flask containing distilled and degassed Et_3N/THF (50 mL/10 mL) were added under nitrogen 97 mg (0.138 mmol) of diclorobis(triphenyl) phosphine palladium II, 13 mg (0.069 mmol) of CuI and 3000 mg (4.6 mmol) of **5**. The mixture was heated to 50°C for 10 min and then cooled to 0°C. Later 1090 mg (4.6 mmol) of **6** in 5 mL of THF was added via syringe. The mixture was left to reach the room temperature and to react for 48 h. After filtering the ammonium salt, the solvent was evaporated and the crude product was purified by column chromatography (SiO_2, Hexanes: CH_2Cl_2, 2:1 v/v, rf = 0.4). Beige powder, 65% yield (2.28 g). mp = 51–52 °C. ^{1}H NMR (300 MHz, $CDCl_3$) δ (ppm) 8.06 (d, 2H, H2, H6), 7.58 (d, 2H, H3, H5), 7.47-7.33 (m, 5H, -Bz), 7.11 (s, 1H, H11), 7.02 (s, 1H, H8), 5.38 (s, 2H, -CH2-Bz), 3.98 (t, 4H, -CH_2-α-O-), 1.82 (s, 4H, -CH_2-β-O-), 1.50 (s, 4H, -CH_2-γ-O-), 1.26 (d, 32H, -CH_2-), 0.89 (m, 6H, -CH_3). ^{13}C NMR (300 MHz, $CDCl_3$) δ (ppm) 165.94 (C=O), 154.45 (C12), 149.57 (C9), 136.03 (-CH_2-C[Bz]), 131.49 (C3, C5), 129.74 (C2, C6), 129.48 (C1), 128.73-128.34 (C4, Bz), 118.02 (C8), 117.58 (C11), 114.14

(C10), 112.04 (C7), 93.32 y 88.75 (C≡C), 70.19 y 69.87 (C-α-O), 66.97 ($-CH_2$-Bz), 32.05 (C-β-CH_3), 29.77–29.30 ($-CH_2$-, C-β-O), 26.10 (C-γ-O), 22.82 (C-α-CH_3), 14.25 ($-CH_3$).

Compound (8). Applying the procedure for the Sonogashira cross-coupling: in a two neck flask containing distilled and degassed Et_3N (50 mL) were added under nitrogen 17 mg (0.024 mmol) of diclorobis(triphenyl) phosphine palladium II, 3 mg (0.018 mmol) of CuI and 600 mg (0.79 mmol) of **7**. The mixture was heated at 50 °C for 10 min and then 174 mg (0.79 mmol) of **6** in 5 mL of THF were added by syringe. The mixture was heated at 60 °C for 18 h. After filtering the ammonium salt, the solvent was evaporated and the crude product was purified by column chromatography (SiO_2, CH_2Cl_2, rf = 0.4). Yellow fluorescent powder, 70 % yield (465 mg). mp = 55-57°C. ^{1}H NMR (300 MHz, $CDCl_3$) δ (ppm) 8.04 (d, 2H, H2, H6), 7.57 (d, 2H, H3, H5), 7.49–7.31 (m, 7H, H14, H18 y Bz), 6.99 (s, 2H, H8, H11), 6.88 (d, 2H, H15, H17), 5.36 (s, 2H, -CH2-Bz), 4.15 (t, 2H, ($-CH_2$-α-O-Ar), 4.02 (t, 4H, $-CH_2$-α-O-), 3.86 (t, 2H, $-CH_2$-β-O-Ar), 3.72 (t, 2H, $-CH_2$-α-O-CH_3), 3.58 (t, 2H, $-CH_2$-β-O-CH_3), 3.39 (s, 3H, CH_3-O-), 1.84 (q, 4H, $-CH_2$-β-O-), 1.53 (q, 4H, $-CH_2$-γ-O-), 1.24 (s, 32H, $-CH_2$-), 0.87 (t, 6H, $-CH_3$). ^{13}C NMR (300 MHz, $CDCl_3$) δ (ppm) 165.89 (C=O), 159.01 (C-Ar[O-((CH_2)$_2$-O)$_2$-CH_3]), 153.98 (C12), 153.49 (C9), 136.03 (($-CH_2$-C[Bz]), 133.16 (C14, C18), 131.51 (C3, C5), 129.74 (C2, C6), 129.37 (C1), 128.70–128.30 (C4, Bz), 116.99 (C8), 116.73 (C11), 115.79 (10), 115.24 (C7), 114.73 (C15, C17), 112.83 (C13), 95.46-84.82 (C≡C) , 72.05 (C-α-O-CH_3) 70.89 (C-α-O-Ar), 69.58 (C-β-O-CH_3), 67.52 (C-β-O-Ar), 69.70 (C-α-O), 66.93 ($-CH_2$-Bz), 59.15 (CH_3-O), 32.04 (C-β-CH_3), 29.77-29.30 ($-CH_2$-, C-β-O), 26.19 (C-γ-O), 22.82 (C-α-CH_3), 14.26 ($-CH_3$). UV (DMF): λmax = 380 nm, ε = 79200 $M^{-1}cm^{-1}$.

Compound (9). In a 100 mL flask, 80 mg (0.072 mmol) of **8** and 41 mg (0.733 mmol) of dry and pulverized KOH were dissolved in 15 mL of dry toluene. After degassing by bubbling nitrogen into the solution for 45 min, the reaction flask was heated at 110 °C for 120 min under nitrogen atmosphere pressure (using a rubber balloon). After cooling to room temperature, HCl (pH = 2) water solution was added, to obtain a precipitate that was filtered off, washed with ethanol, water and dried. Beige powder, 87% yield (103 mg). mp = 87–89 °C. ^{1}H NMR (300 MHz, $CDCl_3$) δ (ppm) 8.07 (d, 2H, H2, H6), 7.60 (d, 2H, H3, H5), 7.50 (d, 2H, H14, H18), 7.00 (s, 2H, H8, H11), 6.88 (d, 2H, H15, H17), 4.14 (t, 2H, $-CH_2$-α-O-Ar), 4.01 (t, 4H, $-CH_2$-α-O-), 3.86 (t, 2H, $-CH_2$-β-O-Ar), 3.73 (t, 2H, $-CH_2$-α-O-CH_3), 3.58 (t, 2H, $-CH_2$-β–O-CH_3), 3.40 (s, 3H, CH_3-O-), 1.84 (q, 4H, $-CH_2$-β-O-), 1.51 (q, 4H, $-CH_2$-γ-O-), 1.24 (s, 32H, $-CH_2$-), 0.84 (t, 6H, $-CH_3$). ^{13}C NMR (300 MHz, $CDCl_3$) δ (ppm) 158.99 (C-Ar[O-((CH_2)$_2$-O)$_2$-CH_3]), 154.05 (C12), 153.50 (C9), 133.16 (C3, C5, C14, C18), 131.57 (C2, C6), 130.23 (C1), 129.25 (C4), 117.10 (C8), 116.80 (C11), 115.79 (C10), 115.34 (C7), 114.72 (C15, C17), 112.83 (C13), 95.48-84.73 (C≡C), 72.09 (C-α-O-CH_3) 70.85 (C-α-O-Ar), 69.76 (C-α-O), 69.67 (C-β-O-CH_3), 67.53 (C-β-O-Ar), 59.13 (CH_3-O), 32.00 (C-β-CH_3), 29.79-29.46 ($-CH_2$-, C-β-O), 26.16 (C-γ-O), 22.80 (C-α-CH_3), 14.20 ($-CH_3$). UV (DMF): λmax = 374 nm, ε = 21454 $M^{-1}cm^{-1}$.

Compound (12). In a 25 mL round bottomed flask equipped with septa and a magnetic stirrer were added, 70 mg (0.086 mmol) of **9**, 50 mg (0.13 mmol) of the activating agent DTBP **10** and 1.5 mL of dry N-methyl-2-pirrolidinone. The solution was kept stirring for 5 min until all the reactants were dissolved. Then 9 mg (12 μL, 0.086 mmol) of distilled Et_3N and 24 mg (0.086 mmol) of 4-aminophenyl α–D-mannopyranoside **11** dissolved in 1.5 mL of N-methyl-2-pyrrolidinone were added with a syringe. The reaction was left stirring for 4 days at room temperature. After this time, the solution was precipitated in a mixture of ethanol/water (3:7 v/v) and stirred for 20 min. The precipitate was recovered by centrifugation and purified by column chromatography (SiO_2, $CHCl_3$:CH_3OH, 8:2 v/v, rf = 0.5). Yellow fluorescent powder, 75 % yield (70 mg). mp = 171–173 °C. ^{1}H NMR (300 MHz, DMSO-d6) δ (ppm) 10.25 (s, 1H, NH), 8.00 (d, 2H, H2, H6), 7.68 (d, 2H, H-meta-ArMan), 7.62 (d, 2H, H3, H5), 7.42 (d, 2H, H14, H18), 7.15 (s, H, H8), 7.12 (s, 1H, H11), 7.08 (d, 2H, H-ortho-ArMan), 6.98 (d, 2H, H15, H17), 5.33 (s, 1H, Hanomeric), 5.04 (d, 1H, HOCH), 4.86 (d, 1H, HOCH) 4.78 (d, 1H, HOCH), 4.49, (t, 1H, $HOCH_2$) 4.11 (t, 2H, $-CH_2$-α-O-Ar), 4.02 (d, 4H, $-CH_2$-α-O-), 3.82 (s, 1H, H2-Man), 3.73 (t, 2H, $-CH_2$-β-O-Ar), 1.73 (s, 4H, $-CH_2$-β-O-), 1.48 (s, 4H, $-CH_2$-γ-O-), 1.18 (d, 32H, $-CH_2$-), 0.8 (m, 6H, $-CH_3$). δ (ppm). ^{13}C NMR (300 MHz, DMSO-d6) δ (ppm) 164.17 (C=O), 159.41 (C-Ar[O-((CH_2)$_2$-O)$_2$-CH_3]), 153.85 (C12), 153.45 (C9), 153.24, (C-Ar[O-Man], 135.09 (C-Ar[NHCO]), 133.93 (C1), 133.30 (C14, C18), 131.56 (C3, C5), 128.54 (C2, C6), 126.15 (C4), 122.32 (C-meta-Ar[Man]), 117.54 (C-

ortho-Ar[Man]), 117.09 (C8), 116.89 (C11), 115.44 (C15, C17), 115.13 (C7), 114.74 (C10), 112.70 (C13), 99.76 (C-anomeric), 95.69-85.35 (C≡C), 75.43 (C2-Man), 71.85 (C-α-O-CH_3), 71.26 (C5-Man), 70.69 (C3-Man), 70.30 (C-α-O-Ar), 69.37 (C-α-O, C-β-O-CH_3), 67.88 (C-β-O-Ar), 67.27 (C4-Man), 61.59 (C6-Man), 58.63 (CH3-O), 31.88 (C-β-CH_3), 29.68-29.32 (-CH_2-, C-β-O), 26.13 (C-γ-O), 22.68 (C-α-CH_3), 14.51 (-CH_3). UV (DMF): λmax = 376 nm, ε = 26480 $M^{-1}cm^{-1}$.

Compound (13). Applying the procedure for the Sonogashira cross-coupling: In a two neck flask containing distilled and degassed Et_3N (30 mL) were added under nitrogen 15 mg (0.021 mmol) of diclorobis(triphenyl) phosphine palladium II, 4 mg (0.021 mmol) of CuI and 500 mg (0.72 mmol) of **5**. The mixture was heated at 50 °C for 10 min and then 340 mg (1.43 mmol) of **6** in 5 mL of dry and nitrogen bubbled THF were added by syringe. The mixture was heated at 60 °C for 18 h. After filtering the ammonium salt, the solvent was evaporated and the crude product was re-dissolved in 4 mL of CH_2Cl_2 and precipitated in methanol. Then, the crude product was purified by column chromatography (SiO_2, hexanes:CH_2Cl_2, rf = 0.4). Yellow fluorescent powder, 87% yield (570 mg). mp = 105–107 °C. ^1H NMR (300 MHz, $CDCl_3$) δ (ppm) 8.07 (d, 4H, H2, H6, H15, H17), 7.59 (d, 4H, H3, H5, H14, H18), 7.47–7.33 (m, 10H, Bz), 7.03 (s, 2H, H8, H11), 5.38 (s, 4H, -CH_2-Bz), 4.04 (t, 4H, -CH_2-α-O-), 1.86 (q, 4H, -CH_2-β-O-), 1.55 (q, 4H, -CH_2-γ-O-), 1.24 (s, 32H, -CH_2-), 0.88 (t, 6H, -CH_3). ^{13}C NMR (300 MHz, $CDCl_3$) δ (ppm) 165.96 (C=O), 153.90 (C9, C12), 136.01 (-CH_2-C[Bz]), 131.56 (C3, C5, C14, C18), 129.75 (C2, C6, C15, C17), 129.54 (C1, C16), 128.73 (C4, C13), 128.43-128.34 (Bz), 116.94 (C8, C11), 114 (C7, C10), 94.42 y 89.14 (-C≡), 69.69 (C-α-O), 66.98 (-CH_2-Bz), 32.02 (C-β-CH_3), 29.74–29.41 (-CH_2-, C-β-O), 26.16 (C-γ-O), 22.80 (C-α-CH_3), 14.24 (-CH_3). UV (DMF): λmax = 385 nm, ε = 49800 $M^{-1}cm^{-1}$.

Compound (14). In a 100 mL flask, 180 mg (0.194 mmol) of **13** and 110 mg (1.94 mmol) of dry and pulverized KOH were dissolved in 25 mL of dry toluene. After degassing by bubbling nitrogen into the solution for 45 min, the reaction flask was heated at 110 °C for 120 min under nitrogen atmosphere pressure (using a rubber balloon). After cooling to room temperature, HCl (pH = 2) water solution was added to obtain a precipitate that was filtered off, washed with ethanol, water and then dried. The crude product was re-dissolved in THF and precipitated twice in $CHCl_3$. Beige powder, 90% yield (120 mg). mp = 244–247 °C.^1H (300 MHz, THF-d8) δ (ppm) 8.02 (d, 4H, H2, H6, H15, H17), 7.57 (d, 4H, H3, H5, H14, H18), 7.11 (s, 2H, H8, H11), 4.06 (t, 4H, -CH_2-α-O-), 1.85 (q, 4H, -CH_2-β-O-), 1.58 (q, 4H, -CH_2-γ-O-), 1.27 (s, 32H, -CH_2-), 0.88 (t, 6H, -CH_3). ^{13}C NMR (300 MHz, THF-d8) δ (ppm) 166.08 (C=O), 153.97 (C9, C12), 131.09 (C3, C5, C14, C18), 130.48 (C1, C16), 129.63 (C2, C6, C15, C17), 127.88 (C4, C13), 116.57 (C8, C11), 113.95 (C7, C10), 93.92 y 88.79 (C≡C), 69.18 (C-α-O), 31.97 (C-β-CH_3), 29.77–29.42 (-CH_2-, C-β-O), 26.17 (C-γ-O), 22.66 (C-α-CH_3), 13.54 (-CH_3). UV (DMF): λmax = 378 nm, ε = 18405 $M^{-1}cm^{-1}$

Compound (15). In a 25 mL round bottomed flask equipped with septa and a magnetic stirrer were added, 75 mg (0.103 mmol) of **14**, 118 mg (0.309 mmol) of the activating agent DTBP **10** and 1.5 mL of dry N-methyl-2-pirrolidinone. The solution was kept stirring for 5 min until all the reactants were dissolved. Then 21 mg (29 μL, 0.206 mmol) of distilled Et_3N and 56 mg (0.206 mmol) of 4-aminophenyl α–D-mannopyranoside **11** dissolved in 2 mL of N-methyl-2-pirrolidinone were added with a syringe. The reaction was left stirring for 4 days at room temperature. After this time, the solution was precipitated in a mixture of ethanol/water (10:0.5 v/v) and stirred for 20 min. The precipitate was recovered by centrifugation, re-dissolved in NMP and precipitated twice in $CHCl_3$. Pale yellow powder, 77% yield (98 mg). mp = 244–247 °C. ^1H NMR (300 MHz, DMSO-d6) δ (ppm) 10.26 (s, 2H, NH), 8.01 (d, 4H, H2, H6,H15, H17), 7.66 (t, 8H, H3, H5,H14, H18 y 4H-meta-Ar[Man]), 7.23 (s, 2H, H8, H11), 7.09 (d, 4H, H-ortho-Ar[Man]), 5.33 (s, 2H, H anomeric), 5.04 (d, 2H, HOCH), 4.85 (d, 2H, HOCH), 4.77 (d, 2H, HOCH), 4.49, (t, 2H, $HOCH_2$), 4.07 (s, 4H, -CH_2-α-O-), 3.82 (s, 2H, H2Man), 1.75 (s, 4H, -CH_2-β–O-), 1.51 (s, 4H, -CH_2-γ-O-), 1.35 (s, 32H, -CH_2-), 0.79 (m, 6H, -CH_3). RMN 13C (300 MHz, DMSO-d6) δ (ppm) 164.78 (C=O), 153.84 (C9, C12), 153.28 (C-Ar[Man]), 135.22(C-Ar[NHCO]), 133.93 (C1, C6), 131.61 (C3, C5, C14, C18), 128.55 (C2, C6, C15, C17), 126.06 (C4, C13), 122.35 (C-meta-Ar[Man]), 117.57 (C-ortho-Ar[Man]), 117.27 (C8, C11), 113.80 (C7, C10), 99.84 (C anomeric), 94.76 y 88.85 (C≡C), 75.43 (C2-Man), 71.29 (C5-Man), 70.71 (C3-Man), 69.49

(C-α-O), 67.34 (C4-Man), 61.64 (C6-Man), 31.84 (C-β-CH_3), 29.66-29.42 (-CH_2-, C-β-O), 26.13 (C-γ-O), 22.64 (C-α-CH_3), 14.46 (-CH_3). λmax = 381 nm, ε = 91200 $M^{-1}cm^{-1}$.

II. *E. coli* growth and Microscopic Characterization, Agglutination Tests and additional LSCM Images

***E.coli* growth and microscopic characterization.** The two *Escherichia coli* strains (ORN 178 and ORN 208), kindly provided by Professor Orndorf (University of North Carolina) [3], and that obtained from a local hospital of Saltillo, Mexico (SS3) were cultivated under designed grow conditions to express Type 1 fimbriae. Cells were inoculated in 75 mL of Luria broth and incubated under static conditions at 37 °C for 48 h. The Luria broth was prepared by dissolving 750 mg of Bacto peptone, 380 mg of yeast extract, 750 mg of NaCl, 75 mg of glucose and 75 mg of $MgCl_2 \cdot 6H_2O$ in 75 mL of distilled water. The growth medium was sterilized, as well as the rest of the solutions and materials used in microbiological assays, at 121 °C for 30 min. Then, a further inoculation was performed by adding 2 mL of the first culture to 75 mL of fresh Luria broth. Later, 15 mL of the cultures were centrifuged at 13000 rpm for 3 min and the cells were suspended in 15 mL of water, centrifuged and washed. This procedure was repeated three times and the biomass was finally suspended in distilled water to obtain a cell number of about 10^8 CFU/mL. The number of viable cells was determined by conventional colony counting on an agar plate.

The microscopic characterization was carried out by Scanning Electron Microscopy (SEM) and Transmission Electron Microscopy (TEM). For SEM characterization, to a suspension of *E.coli* in distilled water was added an equal volume of 2 percent (w/v) of potassium phosphotungstate and manually stirred. Then, a drop of this mixture was put on a carbon grid, air dried and immediatelly observed under a SM510 TOPCON microscope, or on a QUANTA 200 3D, operating at 30 kV and working distance of 5 mm. The specimens were examined at a magnification of 12000X. A typical image is shown in Figure S1.

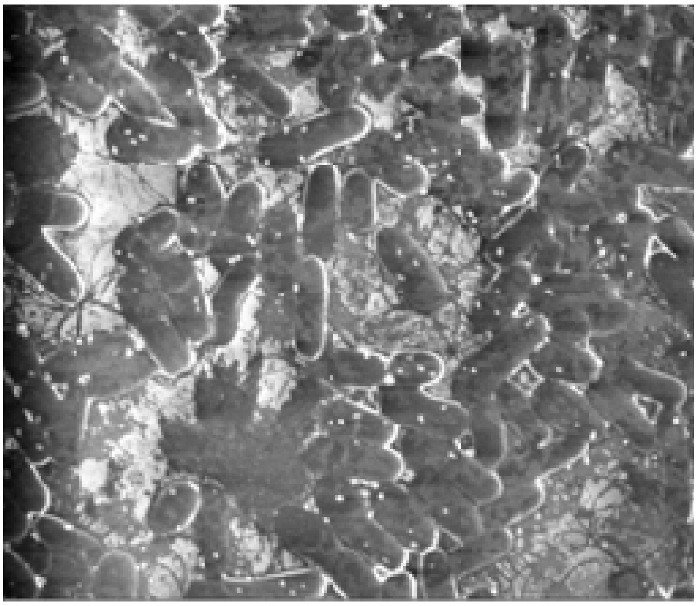

Figure S1. Scanning electron microscopy images of a stained *E. coli* from a local hospital (SS3) at 12000X of magnification.

For TEM studies, the bacterial suspensions were deposited by casting from water on lacey carbon grids and were examined in a TEM mode in an FEI-TITAN-30 kV field emission gun microscope, which has a symmetrical condenser-objective lens type S-TWIN (with an spherical aberration Cs = 1.25 mm).

Agglutination tests. The fimbriae 1 was extracted from cell as described by Evans et al. [4]. Briefly, after extraction in a mixer, bacteria were removed by centrifugation and fimbriae 1 were purified using an ultrafiltration pressure cell as described by Khan et al. [5]. The success in fimbriae 1 isolation was corroborated by an agglutination test performed as follows: on a microscope slide, 10 µL of bacterial suspension (10^{10} cells per ml in PBS) were mixed with the yeast cells (10^{6} cells per ml in PBS). Mannose (250 mM) was used as inhibitor. The tests were followed by optical microscopy using a Leica ATC 2000 microscope and the corresponding optical microscopy images are shown in Figures S2 (agglutination) and S3 (inhibition) together with the sketches of the expected mechanisms.

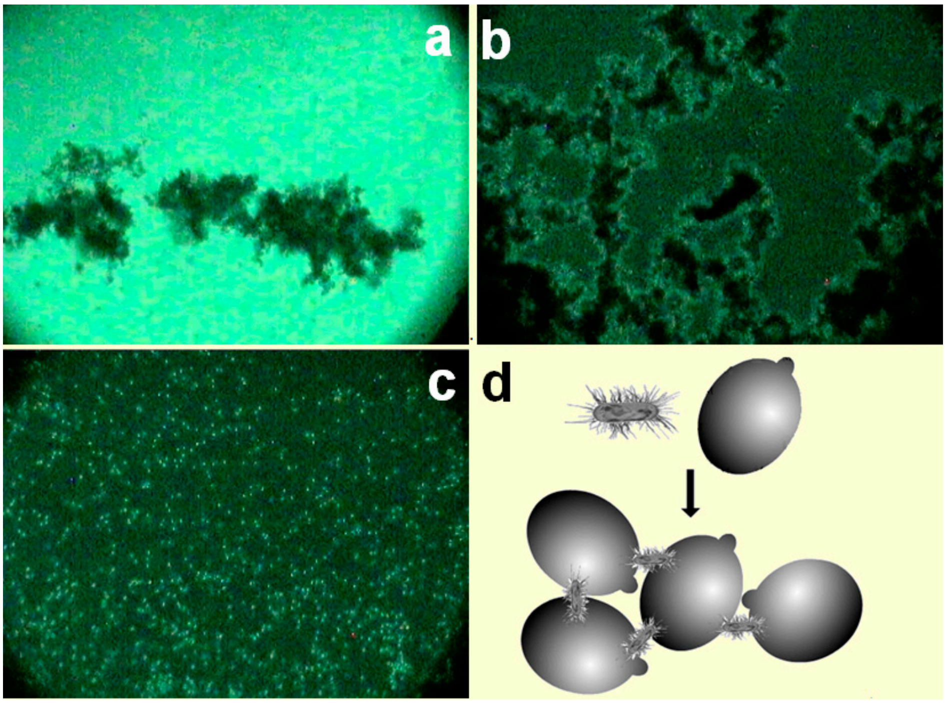

Figure S2. Optical microscopy images (X 200 of magnification) of agglutination test of different *E. coli strains* with *Saccharomyces cerevisiae*: (**a**) SS3, (**b**) ORN178 and (**c**) ORN208. Agglutination is due to the presence of fimbriae 1, as sketched in (**d**).

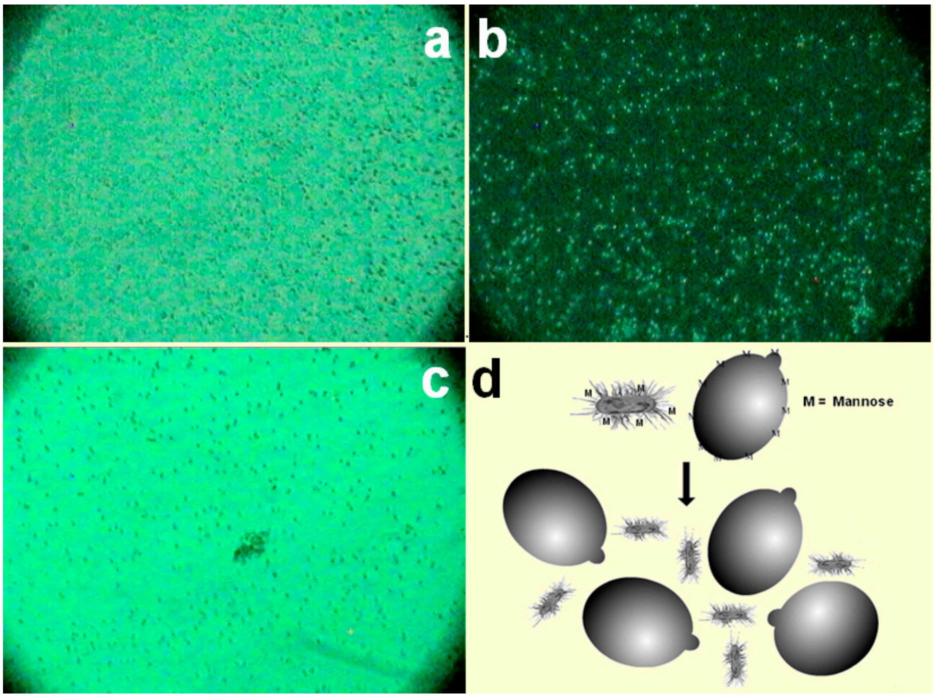

Figure S3. Optical microscopy images (X 200 of magnification) of inhibition of the agglutination of different strains of *E. coli* with *Saccharomyces cerevisiae* by using mannose as inhibitor of: (**a**) SS3, (**b**) ORN178 and (**c**) ORN208. Inhibition of agglutination is due to the presence of mannose in the medium as sketched in (**d**).

Additional LSCM images. As a control, LSCM studies were performed with SS3 and ORN208 *E.coli* strains and a glycol substituted phenyleneethynylene (3BEgly-H, [6]) following the same procedure that was reported in the main text. Typical LSCM images are shown in Figure S4.

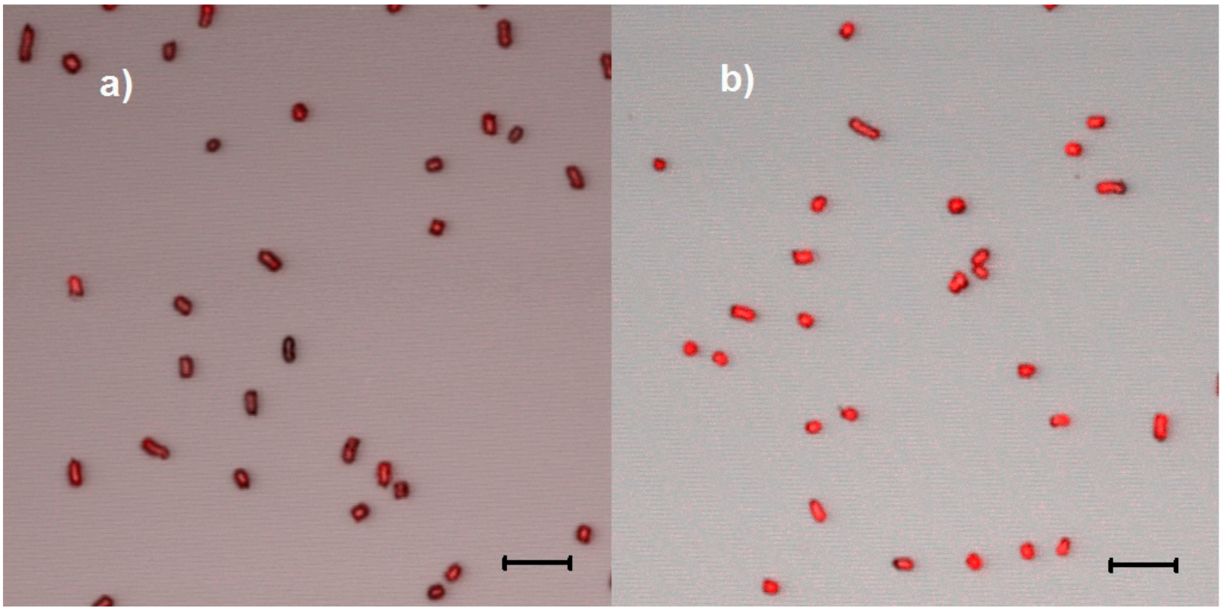

Figure S4. LSCM image of (**a**) SS3 and (**b**) ORN208 *E.coli* bacteria with a glycol substituted phenyleneethynylene (3BEgly-H, [6]. Scale bar corresponds to 5 µm.

III. SPR Chip Preparation

Gold is so far the most popular metal considered for SPR applications as surface plasmons can be efficiently excited with radiations belonging to the VIS-NIR region of the spectrum [7], it possesses a high chemical stability and offers the possibility to be promptly functionalized with receptors through self-assembly monolayers (SAM) technique, exploiting well known chemistries based on its affinity with thiols or amines. In our case, the compound **15**, the specific receptor for the type 1 fimbriae of bacteria, doesn't possess functional groups with affinity to gold. Indeed, we verified that after its deposition on the gold-coated glass, the film of **15** was easily removed with solvent, water or even just with handling, confirming the absence of a tight interaction with the surface. Moreover, gold interacts with proteins. Indeed we performed a control assay of gold in the presence of glucose oxidase, obtaining a SPR curve typical of an affinity process (Figure S5).

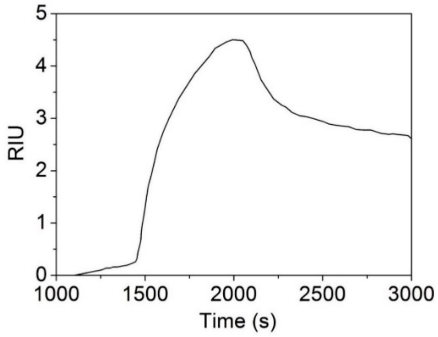

Figure S5. SPR curve for **15** deposited on gold coated glass in presence of glucose oxidase.

Silver is the other metal widely used for propagative SPR sensing, but undergoes similar problems to gold, while copper is quite rarely used for its scarce SP guidance capability. Aluminum is able to support propagative surface plasmons even in the UV region of the spectrum, but its resonant response is not optimal because it exhibits a dielectric constant with a considerable imaginary part in the VIS-NIR region. This drawback is somehow counterbalanced by a useful chemical property. As is well known, freshly deposited aluminum almost immediately undergoes a surface oxidation that leads to the formation of a transparent layer of alumina that stabilizes the deposition against chemical agents. Moreover, alumina is a common adsorbent for thin layer chromatography (TLC) of organic compounds. For these reasons, we selected aluminum as the best material trading off between the sensing performances and the chemical affinity with oligomer **15**.

To characterize the sensing chip, we first observed by LSCM the deposit of the drop-casted compound onto a sample with metallized surface. A typical picture of the deposit is represented in Figure S6. It can be observed that the oligomer does not form a continuous film but rather homogenous isolated domains with few agglomerates. Nevertheless, as the interrogation laser spot has a diameter of ~1 mm, a statistically relevant number of domains (approximately 10^3/mm^2) is illuminated at the same time, and a spatially non fluctuating response is obtained as result of the effective signal averaging on the interrogation area.

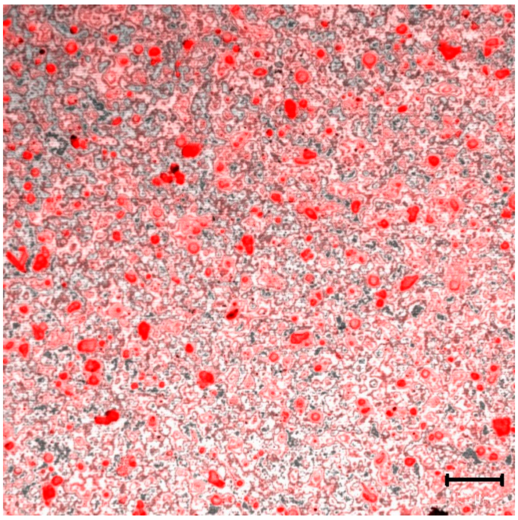

Figure S6. LSCM image of an aluminum substrate coated with **15**. Scaled bar is 50 μm.

IV. Additional SPR Results

SPR biosensing was also performed with SS3 *E. coli* with CFU/mL between 10^6 and 10^9 (Figure S7). Experiments performed with bacteria concentrations lower than 10^6 CFU/mL did not give appreciable response.

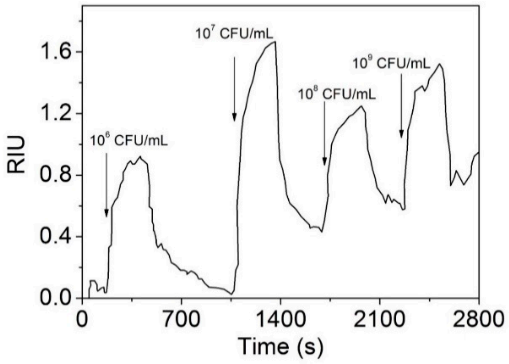

Figure S7. SPR assays of **15** sensor chip to *Escherichia coli* SS3 strain.

References

1. Castruita, G.; Arias, E.; Moggio, I.; Pérez, F.; Medellin, D.; Torres, R.; Ziolo, R.; Olivas, A.; Giorgetti, E.; Muniz-Miranda, M. *J Mol Struct* **2009**, *936*, 177–186.
2. García, L.A.; Arias, E.; Moggio, I.; Romero, J.; Ledezma, A.; Ponce, A.; Pérez. O. *Polymer* **2011**, *52*, 5326–5334.
3. Harris, S.L.; Spears, P.A.; Havell, E.A.; Hamrick, T.S.; Horton, J.R.; Orndorff, P.E. Characterization of *Escherichia coli* type 1 pilus mutants with altered binding specificities. *J. Bacteriol.* **2001**, *183*, 4099–4102.
4. Evans, D.G.; Evans, D.J.; Clegg, S.; Pauley, J.A. Purification and characterization of the CFA/I antigen of enterotoxigenic *Escherichia coli*. *Infect Immun* **1979**, *25,* 738–748.
5. Khan, A.S.; Kniep, B.; Oelschlaeger, T.A.; Van Die, I.; Korhonen, T.; Hacker, J. Receptor structure for F1C fimbriae of uropathogenic *Escherichia coli*. *Infect Immun.* **2000**, *68*, 3541–3547.

6. Meza, D.; Arias, E.; Moggio, I.; Romero, J.; Mata, J.M.; Jiménez-Barrera, R.M.; Ziolo, R.; Rodríguez, O.; Ottonelli, M. Synthesis and photophysical and supramolecular study of π-conjugated (diethylene glycol methyl ether) benzoateethynylene oligomers and polymers. *Polym Chem*. **2015**, *6*, 1639–1648.
7. Pitarke, J.M.; Silkin, V.M.; Chulkov, E.V.; Echenique, P.M. Theory of surface plasmons and surface-plasmon polaritons. *Rep. Prog. Phys.* **2007**, *70*, 1–87.
